# Impact of drug burden index on adverse health outcomes in Irish community-dwelling older people: a cohort study

**DOI:** 10.1186/s12877-019-1138-7

**Published:** 2019-04-29

**Authors:** Catherine J. Byrne, Caroline Walsh, Caitriona Cahir, Kathleen Bennett

**Affiliations:** Division of Population Health Sciences, Royal College of Surgeons in Ireland, Dublin, Ireland

**Keywords:** Drug burden index, Anticholinergic and sedative medications, Older people, Health outcomes, Potentially inappropriate prescribing

## Abstract

**Background:**

The Drug Burden Index (DBI) quantifies exposure to medications with anticholinergic and/or sedative effects. A consensus list of DBI medications available in Ireland was recently developed for use as a DBI tool. The aim of this study was to validate this DBI tool by examining the association of DBI score with important health outcomes in Irish community-dwelling older people.

**Methods:**

This was a cohort study using data from The Irish Longitudinal Study on Ageing (TILDA) with linked pharmacy claims data. Individuals aged ≥65 years participating in TILDA and enrolled in the General Medical Services scheme were eligible for inclusion. DBI score was determined by applying the DBI tool to participants’ medication dispensing data in the year prior to outcome assessment. DBI score was recoded into a categorical variable [none (0), low (> 0 and < 1), and high (≥1)]. Outcome measures included any Activities of Daily Living (ADL) impairment, any Instrumental Activities of Daily Living (IADL) impairment, any self-reported fall in the previous 12 months, any frailty criterion met (Fried Phenotype measure), quality of life (QoL) score (CASP-19 [Control Autonomy Self-realisation Pleasure] measure), and healthcare utilisation (any hospital admission and any emergency department (ED) visit) in the previous 12 months. Statistical analyses included multivariate logistic and linear regression models controlling for potential confounders.

**Results:**

61.3% (*n* = 1946) of participants received at least one DBI prescription in the year before their outcome assessment. High DBI exposure (DBI score ≥ 1) vs none was significantly associated with impaired function (ADL impairment adjusted OR 1.89, 95% CI 1.25, 2.88; IADL impairment adjusted OR 2.97, 95% CI 1.91, 4.61), self-reported falls (adjusted OR 1.50, 95%CI 1.03, 2.18), frailty (adjusted OR 1.74, 95% CI 1.14, 2.67), and reduced QoL (β = − 1.84, 95%CI -3.14, − 0.54). There was no significant association between DBI exposure and healthcare utilisation.

**Conclusions:**

The findings validate the use of the DBI tool for predicting risk of functional impairment, falls, frailty and reduced QoL in older people in Ireland, and may be extended to other European countries. Integration of this tool into routine practice may be an appropriate step forward to improve outcomes in older people.

## Background

An area of specific concern in terms of potentially inappropriate prescribing in older people is the prescribing of medications with anticholinergic and/or sedative properties. These medications are used to treat a range of conditions that occur commonly in later life, including urinary incontinence, sleep disturbances, mental illness, pain, and gastrointestinal disorders [1]. In older patients, with multiple comorbidities, this may result in an additive anticholinergic and sedative burden.

The Drug Burden Index (DBI) is a novel risk assessment tool to quantify older individuals’ cumulative exposure to medications with clinically significant anticholinergic and/or sedative effects [2]. A growing number of studies conducted in older aged populations in several different countries have demonstrated an association between higher DBI scores – that is, greater exposure to anticholinergic and/or sedative medications – and a range of adverse outcomes including poorer physical function, falls, frailty, lower quality of life (QoL), and healthcare utilisation [3].

A consensus list of DBI medications relevant to Ireland, and their corresponding minimum daily dosages in older people, was previously developed and applied to a national pharmacy claims database in Ireland [4]. This involved using the Irish DBI list in conjunction with the original DBI formula [2], referred to as the DBI tool, to determine an individual’s DBI score. The relationship between DBI score and health outcomes in older aged people living in Ireland has not previously been examined. The aim of this study was to validate this DBI tool, by examining the association of DBI score with important health outcomes in a representative cohort of Irish community-dwelling older people using a linked data resource.

## Methods

### Study design, setting and participants

This cohort study used data from Wave 1 of The Irish Longitudinal Study on Ageing (TILDA), which has been described in detail elsewhere [5]. For TILDA Wave 1, data were collected from a representative sample of the Irish community-dwelling population, aged 50 years and older, from October 2009 to February 2011, through a computer-assisted personal interview, a self-completed questionnaire, and a nurse-led health assessment [5]. Written informed consent to participate in TILDA was provided by each participant. Consent was also provided by participants to the use of their administrative pharmacy claims data from the Health Service Executive Primary Care Reimbursement Service (HSE-PCRS). Ethical approval for TILDA was granted by the Faculty of Health Sciences Ethics Committee, Trinity College Dublin, which included secondary analysis of collected data and provision for linkage to participants’ GMS dispensing information. Permission to use the HSE-PCRS data for the purposes of this research was granted by the HSE-PCRS.

In the present study, participants were included if they were aged ≥65 years at their TILDA Wave 1 interview, were enrolled in the General Medical Services (GMS) scheme, and presented a GMS identifier which could be linked to their pharmacy claims data [6]. The GMS scheme is a form of public health cover in Ireland, with eligibility for the scheme based on means testing. The GMS scheme provides mainly free health services to eligible persons. A small monthly co-payment for prescription items was introduced in October 2010. The GMS scheme is the single largest pharmacy claims dataset in Ireland, covering approximately 40% of the general Irish population. However, a considerably higher income threshold for eligibility is applied for people aged over 70 years, with approximately 96% of this age group being eligible in 2011 [7, 8].

In the HSE-PCRS pharmacy claims database, medicines are coded using the World Health Organisation Anatomical Therapeutic Chemical (ATC) classification system [9]. For each participant in this study, details of prescribed medicines that were dispensed were extracted from the HSE-PCRS pharmacy claims database from two years before the date of their TILDA Wave 1 interview up to the interview date. All data were anonymised after linkage.

### Medication exposure

The DBI tool was applied to participants’ medication dispensing data to determine DBI exposure [4]. DBI medications (with dose information) were identified using relevant ATC codes.

**Fig. 1:**
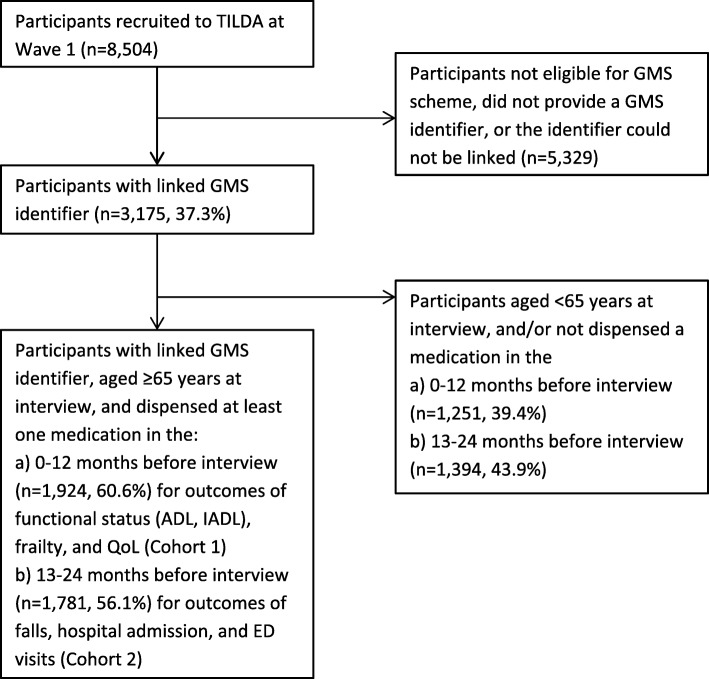
Flow diagram of study participants from The Irish Longitudinal Study on Ageing (TILDA) cohort aged 65 years and over. ADL, activities of daily living; ED, hospital emergency department; GMS, General Medical Services; IADL, instrumental activities of daily living; QoL, quality of life

Total DBI exposure for each participant was calculated as the sum of exposure to any DBI medication dispensed in the 12 months before the time-period specified for outcome assessment. Outcomes included in this study were either assessed at the time of interview or over the 12-month period preceding the interview. As GMS eligibility may change over time, 2 cohorts of participants were included in this study – Cohort 1 included eligible participants in the year preceding the interview date, and Cohort 2 included eligible participants in the year preceding one year before the interview date, to account for varying time windows for outcome assessment. Outcomes relating to an individual’s condition at the time of the interview included functional status, frailty and QoL. For these outcomes, DBI exposure was determined from 0 to 12 months prior to the interview date (Cohort 1) (Fig. 1). Outcomes relating to an individual’s condition over the 12-month period preceding the interview included self-reported falls and healthcare utilisation. For these outcomes, DBI exposure was determined from 13 to 24 months prior to the interview date (Cohort 2) (Fig. 1).

DBI score for each patient was calculated using the following formula [2]:

$$ DBI=\sum D/\left(\delta +D\right) $$

where *D* is the patient’s daily dose of a DBI medication and *δ* is the minimum recommended daily dose for that drug. For each DBI medication, the daily dose taken by the individual patient was estimated by multiplying the strength and total quantity dispensed over the 12-month period, and then dividing by 365 days to normalise to an average daily dose. The scores for each DBI medication taken by the individual patient were summed to give that patient’s total DBI score. The total DBI score was then recoded into a categorical variable [none (0), low (> 0 and < 1), and high (≥1)].

### Outcomes

The Activities of Daily Living (ADL) scale [10], and the Instrumental Activities of Daily Living (IADL) scale [11], were used to assess functional status. For each of these scales, disability was defined as ≥1 self-reported inability to perform a listed activity. Falls were defined as ≥1 self-reported fall in the previous 12 months. Frailty was assessed using the Fried Phenotype measure [12]. Five criteria based on the participant’s objective and self-reported measures were used to construct this frailty measure: gait speed, exhaustion, physical inactivity, unintentional weight loss, and grip strength [12]. Participants were classified as frail if they met ≥1 frailty criterion. QoL was assessed using the CASP-19 (Control Autonomy Self-realisation Pleasure) measure [13]. In this measure, participants rate how often each item describes how they feel, giving a possible range of scores from 0 (worst QoL) to 57 (best QoL). Healthcare utilisation was based on the participant’s self-report of any hospital admission, and any visit to the hospital emergency department (ED) as a patient, in the previous 12 months.

### Covariates

Age, sex, education level, living arrangements, polypharmacy, number of chronic diseases, depression [14], and cognitive function [15], were included as covariates in models for all outcome measures. For healthcare utilisation outcome measures (hospital admission and ED visits), disability, defined as any ADL or IADL difficulty, was also included as a covariate. Details of covariates adjusted for in the multivariate regression models are provided in Table 1.

**Table 1:** Description of covariates adjusted for in multivariate regression models

Variable	Format	Description of categories
Age (in years)	Continuous	N/A
Sex	Binary	Male (reference)
		Female
Education level	Categorical	None or Primary (reference)
		Secondary
		Tertiary
Living arrangements	Binary	Living alone (reference)
		Living with spouse or others
Polypharmacy^a^	Binary	No (reference)
		Yes
Number of chronic diseases^b^	Categorical	0 (reference)
		1
		2
		3 or more
Depression^c^	Categorical	None/mild (reference)
		Moderate
		Severe
Cognitive function^d^	Binary	Normal (MMSE≥25, reference)
		Impaired (MMSE< 25)
Disability^e^	Binary	No (reference)
		Yes (any ADL or IADL difficulty)

^a^Polypharmacy defined as taking > 5 regular medications

^b^The number of doctor-diagnosed chronic conditions reported by participants from the following list: cardiovascular disease (heart attack, heart failure or angina), cataracts, hypertension, high cholesterol, stroke, diabetes, lung disease, asthma, arthritis, osteoporosis, cancer, Parkinson’s disease, peptic ulcer, and hip fracture

^c^Level of depressive symptoms was determined using the Centre for Epidemiological Studies Depression scale [14], based on the participant’s self-completion questionnaire

^d^Cognitive function was determined using the Mini-Mental State Examination (MMSE) [15], based on the participant’s self-completion questionnaire

^e^Disability was defined as at least one self-reported difficulty with any task listed in either the Activities of Daily Living (ADL) scale [10], or the Instrumental Activities of Daily Living (IADL) scale [11]

### Statistical analyses

The associations between the categorised DBI score and outcome measures were analysed using multivariate regression analyses. For the binary outcome measures of function (ADL and IADL), self-reported falls, frailty, hospital admission, and ED visits, logistic regression was used, with results presented as odds ratios (OR) with 95% CI. For QoL, linear regression was used, with results presented as β coefficients with 95% CI. Participants with missing data for any outcome, exposure or covariate were excluded from that analysis.

All significance tests were two-tailed. Statistical significance was set at *P* < 0.05, after adjustment for a false discovery rate of 5% [16]. Data were analysed using Stata v 15 (StataCorp, College Station, TX, USA).

## Results

1924 participants, and 1781 participants, were included in the cohorts relating to 0–12 months (Cohort 1), and 13–24 months (Cohort 2), before the TILDA interview, respectively. A flow diagram detailing the inclusion and exclusion of study participants is provided in Fig. 1. The demographic and clinical characteristics of participants included in each cohort are provided in Table 2.

**Table 2:** Characteristics of participants included in Cohort 1 (0–12 months before interview) and Cohort 2 (13–24 months before interview)

Characteristic	Cohort 1 (*n* = 1924)	Cohort 2 (*n* = 1781)
Age (years, mean (SD))^a^	75.0 (6.1)	75.3 (6.1)
Female sex (*n* (%))	1052 (54.7)	977 (54.9)
Education level (*n* (%))^b^		
Primary	994 (51.7)	939 (52.8)
Secondary	602 (31.3)	553 (31.1)
Tertiary	326 (17.0)	287 (16.1)
Living alone (*n* (%))	679 (35.3)	652 (36.6)
Polypharmacy (*n* (%))^c^	774 (40.6)	728 (41.3)
No. of chronic diseases (*n* (%))^d^		
0	181 (9.4)	154 (8.6)
1	387 (20.1)	361 (20.3)
2	475 (24.7)	435 (24.4)
3+	881 (45.8)	831 (46.7)
Depression (*n* (%))^e^		
None/mild	1087 (57.5)	985 (56.4)
Moderate	579 (30.7)	547 (31.3)
Severe	223 (11.8)	215 (12.3)
Cognitive impairment (*n* (%))^f^	178 (9.3)	173 (9.7)
Disability (*n* (%))^g^	397 (20.6)	382 (21.5)
Drug Burden Index score		
Mean (SD)	0.63 (0.71)	0.64 (0.70)
Median (IQR)	0.44 (0.07–0.88)	0.46 (0.08–0.91)
Drug Burden Index groups (*n* (%))		
0	727 (37.8)	706 (39.6)
> 0 to < 1	934 (48.5)	850 (47.7)
≥ 1	263 (13.7)	225 (12.6)

^a^Missing for 1 participant (0.05%) in Cohort 1

^b^Missing for 2 participants (0.10%) in Cohort 1, and 2 participants (0.11%) in Cohort 2

^c^Taking > 5 regular medications. Missing for 17 participants (0.88%) in Cohort 1, and 18 participants (1.01%) in Cohort 2

^d^Doctor-diagnosed chronic conditions from the following list: cardiovascular disease (heart attack, heart failure or angina), cataracts, hypertension, high cholesterol, stroke, diabetes, lung disease, asthma, arthritis, osteoporosis, cancer, Parkinson’s disease, peptic ulcer, and hip fracture

^e^Based on the Centre for Epidemiological Studies Depression scale [14]. Missing for 35 participants (1.82%) in Cohort 1, and 34 participants (1.91%) in Cohort 2

^f^Mini-Mental State Examination (MMSE) score < 25 [15]

^g^At least one difficulty with any task listed in either the Activities of Daily Living (ADL) scale [10] or the Instrumental Activities of Daily Living (IADL) scale [11]

Overall, 62.2% (1197) of participants in Cohort 1, and 60.4% (1075) of participants in Cohort 2, received at least one prescription for a DBI medication in the prior year. Further details of DBI exposure are provided in Table 2.

Table 3 summarises the association of DBI exposure with patient outcomes. For these analyses, due to missing data, 55 (2.86%) participants were excluded for both the ADL and IADL outcomes, 703 (36.54%) participants were excluded for the frailty outcome, 678 (35.24%) participants were excluded for the QoL outcome, 55 (3.09%) participants were excluded for both the falls and hospital admission outcomes, and 56 (3.14%) participants were excluded for the ED visits outcome.

**Table 3:** Multivariate models showing the associations of DBI score with adverse health outcomes in Irish community-dwelling older people

	ADL disability^a^ *n* = 1869 OR (95% CI)	IADL disability^b^ *n* = 1869 OR (95% CI)	Falls^c^ *n* = 1726 OR (95% CI)	Hospitalisation^d^ *n* = 1726 OR (95% CI)	ED visits^e^ *n* = 1725 OR (95% CI)	Frailty^f^ *n* = 1221 OR (95% CI)	Quality of Life^g^ *n* = 1246 *β* (95% CI)
DBI exposure							
None (reference)	1.00	1.00	1.00	1.00	1.00	1.00	1.00
Low (DBI score > 0 to < 1)	1.40 (1.00, 1.95)	1.38 (0.95, 2.00)	1.40 (1.08, 1.81)*	1.25 (0.94, 1.67)	1.29 (0.98, 1.71)	1.39 (1.06, 1.83)*	−1.55 (−2.38, − 0.73)*
High (DBI score ≥ 1)	1.89 (1.25, 2.88)*	2.97 (1.91, 4.61)*	1.50 (1.03, 2.18)*	1.33 (0.88, 2.01)	1.44 (0.96, 2.15)	1.74 (1.14, 2.67)*	−1.84 (−3.14, − 0.54)*
Age (years)	1.06 (1.03, 1.08)*	1.09 (1.06, 1.12)*	1.02 (1.00, 1.04)	0.99 (0.97, 1.01)	0.99 (0.97, 1.01)	1.10 (1.08, 1.13)*	−0.41 (− 0.11, 0.02)
Sex							
Male (reference)	1.00	1.00	1.00	1.00	1.00	1.00	1.00
Female	0.76 (0.57, 1.00)	1.72 (1.25, 2.36)*	0.98 (0.77, 1.24)	0.82 (0.63, 1.07)	0.85 (0.66, 1.10)	0.90 (0.69, 1.17)	1.12 (0.33, 1.90)*
Education level^h^							
Primary (reference)	1.00	1.00	1.00	1.00	1.00	1.00	1.00
Secondary	0.95 (0.69, 1.31)	0.72 (0.51, 1.02)	1.21 (0.93, 1.58)	1.12 (0.83, 1.49)	0.93 (0.69, 1.23)	0.94 (0.71, 1.24)	0.91 (0.03, 1.78)
Tertiary	1.10 (0.74, 1.62)	0.82 (0.52, 1.28)	1.26 (0.91, 1.74)	1.01 (0.69, 1.46)	1.17 (0.83, 1.65)	0.79 (0.56, 1.12)	1.17 (0.15, 2.18)
Living arrangements							
Living alone (reference)	1.00	1.00	1.00	1.00	1.00	1.00	1.00
Living with others	1.32 (0.98, 1.78)	1.19 (0.87, 1.63)	0.90 (0.70, 1.14)	1.03 (0.78, 1.35)	0.92 (0.71, 1.20)	0.90 (0.69, 1.19)	−0.56 (−1.40, 0.28)
Polypharmacy^i^							
No (reference)	1.00	1.00	1.00	1.00	1.00	1.00	1.00
Yes	1.93 (1.43, 2.61)*	1.75 (1.26, 2.43)*	1.15 (0.89, 1.49)	1.78 (1.33, 2.37)*	1.37 (1.03, 1.81)*	1.83 (1.38, 2.42)*	−1.02 (−1.90, −0.14)*
No. of chronic diseases^j^							
0 (reference)	1.00	1.00	1.00	1.00	1.00	1.00	1.00
1	1.88 (0.75, 4.72)	1.08 (0.46, 2.56)	1.46 (0.86, 2.49)	1.26 (0.65, 2.46)	1.26 (0.70, 2.26)	0.67 (0.40, 1.10)	−0.15 (−1.64, 1.34)
2	3.06 (1.27, 7.36)	1.49 (0.66, 3.34)	1.28 (0.76, 2.15)	2.07 (1.10, 3.88)	1.45 (0.82, 2.54)	0.69 (0.42, 1.12)	−1.05 (−2.50, 0.41)
≥ 3	4.58 (1.93, 10.84)*	2.30 (1.04, 5.02)	1.76 (1.06, 2.93)	1.95 (1.04, 3.66)	1.57 (0.90, 2.74)	0.98 (0.61, 1.59)	−1.80 (−3.26, −0.34)
Depression^k^							
None/mild (reference)	1.00	1.00	1.00	1.00	1.00	1.00	1.00
Moderate	1.61 (1.18, 2.21)*	2.31 (1.65, 3.24)*	1.19 (0.92, 1.54)	0.96 (0.71, 1.29)	0.97 (0.73, 1.29)	1.65 (1.25, 2.18)*	−2.25 (−3.10, −1.39)*
Severe	3.94 (2.69, 5.77)*	3.77 (2.48, 5.71)*	1.75 (1.24, 2.47)*	1.05 (0.70, 1.57)	1.67 (1.16, 2.42)*	3.43 (2.19, 5.37)*	−9.49 (−10.90, −8.07)*
Cognitive function^l^							
Normal (reference)	1.00	1.00	1.00	1.00	1.00	1.00	1.00
Impaired	1.66 (1.10, 2.50)*	1.75 (1.14, 2.70)*	1.13 (0.77, 1.66)	1.08 (0.70, 1.67)	0.87 (0.56, 1.35)	1.85 (1.23, 2.79)*	−0.22 (−1.66, 1.23)
Disability^m^							
No (reference)	–	–	–	1.00	1.00	–	–
Yes	–	–	–	1.37 (1.01, 1.87)	1.32 (0.97, 1.78)	–	–

*P < 0.05 after adjustment for a false discovery rate of 5% [16]

*β* beta coefficient, *ADL* Activities of Daily Living, *DBI* Drug Burden Index, *ED* hospital emergency department, *IADL* Instrumental Activities of Daily Living, *OR* odds ratio

^a^At least one difficulty with any task listed in the Activities of Daily Living (ADL) scale [10]

^b^At least one difficulty with any task listed in the Instrumental Activities of Daily Living (ADL) scale [11]

^c^One or more self-reported falls in the previous 12 months

^d^One or more self-reported hospital admissions in the previous 12 months

^e^One or more self-reported visits to ED as a patient in the previous 12 months

^f^One or more frailty criteria according to the Fried Phenotype measure [12]

^g^CASP-19 (Control Autonomy Self-realisation Pleasure) score [13]

^h^Highest level of education achieved: Primary includes primary school or no formal education; Secondary includes secondary school or high school or equivalent; Tertiary includes university degree or equivalent

^i^Taking > 5 regular medications

^j^Doctor-diagnosed chronic conditions reported by participants from the following list: cardiovascular disease (heart attack, heart failure or angina), cataracts, hypertension, high cholesterol, stroke, diabetes, lung disease, asthma, arthritis, osteoporosis, cancer, Parkinson’s disease, peptic ulcer, and hip fracture

^k^Based on the Centre for Epidemiological Studies Depression scale [14]

^l^Based on the Mini-Mental State Examination (MMSE) score [15]. Normal cognitive function (MMSE score ≥ 25). Impaired cognitive function (MMSE score < 25). ^m^ At least one self-reported difficulty with any task listed in either the Activities of Daily Living (ADL) scale [10] or the Instrumental Activities of Daily Living (IADL) scale [11]

Low DBI exposure (DBI score > 0 and < 1) vs none was significantly associated with self-reported falls (adjusted OR 1.40, 95% CI 1.08, 1.81), frailty (adjusted OR 1.39, 95% CI 1.06, 1.83), and reduced QoL (β = − 1.55, 95% CI -2.37, − 0.73). High DBI exposure (DBI score ≥ 1) vs none was significantly associated with impaired function (ADL impairment adjusted OR 1.89, 95% CI 1.25, 2.88; IADL impairment adjusted OR 2.97, 95% CI 1.91, 4.61), self-reported falls (adjusted OR 1.50, 95%CI 1.03, 2.18), frailty (adjusted OR 1.74, 95% CI 1.14, 2.67), and reduced QoL (β = − 1.84, 95%CI -3.14, − 0.54). There was no significant association between any DBI exposure and healthcare utilisation (hospital admission or ED visits) (Table 3).

## Discussion

This study is the first to investigate the association between DBI exposure and adverse outcomes in older people from the general population of Ireland. We found that high exposure to DBI medications was independently associated with important adverse health outcomes in Irish community-dwelling older people. The findings are particularly relevant given the high prevalence of anticholinergic and/or sedative medication use observed in this population.

In the present study, high DBI exposure was significantly associated with reduced capacity in performing basic (ADL) and more complex (IADL) tasks of daily living. These findings are consistent with those of previous studies, conducted in several different countries, which investigated the impact of DBI exposure on a range of limitations of function in older adults [2, 17–21]. The present findings also concur with previous studies showing an independent association of DBI exposure with a greater risk of falls and fall-related hospitalisations in older people [22–24]. The association of DBI exposure with an increased risk of frailty is consistent with the one previous study of older community-dwelling men living in Australia [25]. The finding of an independent association of DBI exposure with reduced QoL is also consistent with previous studies. However, these previous studies included cohorts of older people living in residential aged care facilities, with a high prevalence of cognitive impairment and dementia, and used health-related QoL measures [26, 27]. In the present study, a 1-unit increase in DBI score (equivalent to exposure to two additional DBI medications at minimum dose), predicted a decrease in the QoL CASP-19 score of approximately 2 points, which equates to a small but statistically significant effect size [28]. A 2-point reduction in CASP score is equivalent to answering two positively worded statements ‘Rarely’ instead of ‘Sometimes’ [29]. Examples of a positively worded statements in the CASP-19 score include “I can do the things I want to do” and “I feel full of energy these days” [13].

The utility of the DBI tool for predicting risk of increased healthcare utilisation, in terms of hospital admission and ED visits, was not supported by the findings of the present study. Several previous studies have investigated the association of DBI exposure and various aspects of healthcare utilisation with inconsistent results. Some studies have shown a significant association between DBI and increased hospital admission rates and longer length of stay [21, 30], but others have not [31, 32]. In the present study, the use of polypharmacy was the main driver of healthcare utilisation. In a previous study of community-dwelling older people living in Finland, increasing number of regular medications and declining function were found to be stronger predictors of hospitalisation than DBI exposure [31].

The associations of increased DBI exposure with impaired function and falls are understandable given the established pharmacological effects of anticholinergic and sedative drugs, such as drowsiness, dizziness, visual disturbance, cognitive and psychomotor performance impairment, and impairment of balance control [19]. It is also plausible that exposure to medicines that increase the DBI might contribute to the decline in function that characterises frailty [33]. In older people, independence and well-being depend on a sufficient level of physical function [21]. Consequently, factors contributing to a decline in physical function may result in lower QoL. It has been shown that impairments in physical function and activity limitations mediate the effect of chronic disease on QoL (CASP-19 measure) [34].

Overall, the risks associated with exposure to DBI medications suggests that these medications should be avoided in older people unless there is a compelling clinical indication. Furthermore, for all outcomes tested, participants with a high DBI score (DBI ≥1) had a greater risk of adverse outcomes than those in the low DBI group (DBI > 0 to < 1). These findings concur with those of previous studies conducted in other countries [2, 18–21, 24, 25, 27]. Therefore, strategies aimed at reducing the number and/or the dose of DBI medications might lead to improved outcomes.

A major strength of this study is the generalizability of the findings as the cohort exemplifies a true representation of the Irish community-dwelling older population. Further strengths include the use of a large sample, which was well characterised using a broad range of epidemiological and clinically validated measures. Pharmacy claims data were employed, which is likely to be more reliable than self-reported medicines use [35]. However, there are limitations inherent to using pharmacy claims data as non-adherence and medications purchased over-the-counter (OTC) cannot be accounted for. Therefore, the DBI score may not reflect all exposure. However, given that GMS patients can obtain most OTC medicines on prescription for a small co-payment, the risk of bias is likely to be minimal and non-differential across the exposure groups.

A fixed 12-month exposure period was used before outcome assessment, which in the case of healthcare utilisation and falls was also over a fixed 12-month period. Therefore, any effect of DBI would have to be sustained beyond the exposure period in order to be detected [29]. This may have resulted in misclassification and bias of the results towards the null hypothesis [30]. Socioeconomic bias towards low income individuals aged 65–70 years may have affected the findings since only approximately 40% of the population in this age group were covered by the GMS scheme. Socioeconomically deprived individuals may be more prone to multimorbidity and the use of DBI medications, which may result in an overestimation of the impact of DBI score on health outcomes. However, socioeconomic bias in those aged > 70 years is expected to be considerably lower as approximately 96% of this population were covered by the GMS [7, 8]. Missing data for the outcomes of frailty and QoL were relatively high, which may have biased our results. We also acknowledge that much of the data were self-reported and, therefore, there may be a degree of misreporting. In addition, healthcare utilisation and falls were based solely on participant recall over a 12-month period, and validation against administrative records was not possible. Whilst every attempt was made to control for potential confounders, there may be residual confounding. Volunteer bias may also have influenced study findings. Finally, no adjustment was made in terms of the severity of co-morbid conditions, which may have had an impact on the findings.

The demonstration of negative associations between DBI scores and established markers of outcomes in older people has important implications for practice. When treating older people, due consideration should be given to the dose and the cumulative exposure of drugs that have anticholinergic or sedative effects, because the higher the exposure the greater the risk of adverse outcomes. This emphasizes the importance of regular medication reviews, so that doctors can contemplate the risks and benefits when prescribing multiple medications [22]. In practice, the DBI may be useful as a screening tool for older patients, to identify those with high exposure who may be suitable for de-prescribing interventions [3].

Intervention strategies aimed at reducing the burden of anticholinergic and sedative medications in this population are clearly needed. Such strategies have been tested in Australia. Nishtala et al. showed that collaborative pharmacist-led medication review can reduce the prescribing of anticholinergic and sedative medications in older people living in care homes, resulting in a significant decrease in the DBI score [36]. Gnjidic et al. found that provision of information about patients’ DBI scores to general practitioners led to decreased DBI scores in 32% of older patients living in retirement villages [37]. However, whether intervening to reduce DBI in older patients improves patient outcomes remains to be seen.

## Conclusions

This study validates the DBI tool against a spectrum of important adverse health outcomes in Irish community-dwelling older people. Using the DBI tool, increasing DBI score was independently associated with a greater risk of functional impairment, self-reported falls, frailty, and reduced QoL. The findings support the use of the DBI tool for predicting risk in older people in Ireland, and possibly other European countries. The findings also highlight the potential value of prescribers minimising DBI exposure in older patients as much as possible to reduce the risk of adverse health outcomes. Incorporation of the DBI tool into routine practice may be an appropriate step forward to assist prescribers in identifying high-risk prescribing and optimising treatment in older people. Future research should focus on interventional studies to determine whether interventions to reduce DBI scores in older people translate into improved outcomes.

## Abbreviations

ADL: Activities of Daily Living
ATC: Anatomical Therapeutic Chemical
CASP-19: Control Autonomy Self-realisation Pleasure measure
DBI: Drug Burden Index
ED: Emergency department
GMS: General Medical Services
HSE-PCRS: Health Service Executive Primary Care Reimbursement Service
IADL: Instrumental Activities of Daily Living
OR: Odds ratio
OTC: Over-the-counter
QoL: Quality of Life
TILDA: The Irish Longitudinal Study on Aging

## References

[1] Kouladjian L, Gnjidic D, Chen TF (2014). Drug burden index in older adults: theoretical and practical issues. Clin Interv Aging.

[2] Hilmer SN, Mager DE, Simonsick EM (2007). A drug burden index to define the functional burden of medications in older people. Arch Intern Med.

[3] Wouters H, van der Meer H, Taxis K (2017). Quantification of anticholinergic and sedative drug load with the drug burden index: a review of outcomes and methodological quality of studies. Eur J Clin Pharmacol.

[4] Byrne CJ, Walsh C, Cahir C (2018). Anticholinergic and sedative drug burden in community-dwelling older people: a national database study. BMJ Open.

[5] Whelan BJ, Savva GM (2013). Design and methodology of the Irish longitudinal study on ageing. J Am Geriatr Soc.

[6] Sinnott SJ, Bennett K, Cahir C (2017). Pharmacoepidemiology resources in Ireland-an introduction to pharmacy claims data. Eur J Clin Pharmacol.

[7] Health Service Executive (2011). Primary care reimbursement service: statistical analysis of claims and payments 2011.

[8] Central Statistics Office. Census of Population 2011 Profile 2 Older and Younger (Accessed 10 March 2018). Available from: https://www.cso.ie/en/census/census2011reports/census2011profile2-olderandyounger/.

[9] WHO Collaborating Centre for Drug Statistics Methodology, ATC/DDD Index 2017. Available from: https://www.whocc.no/atc_ddd_index/.

[10] Katz S, Ford AB, Moskowitz RW (1963). Studies of illness in the aged. The index of ADL: a standardized measure of biological and Psycholsocial function. Jama..

[11] Lawton MP, Brody EM (1969). Assessment of older people: self-maintaining and instrumental activities of daily living. Gerontologist..

[12] Fried LP, Tangen CM, Walston J (2001). Frailty in older adults: evidence for a phenotype. J Gerontol A Biol Sci Med Sci.

[13] Hyde M, Wiggins RD, Higgs P (2003). A measure of quality of life in early old age: the theory, development and properties of a needs satisfaction model (CASP-19). Aging Ment Health.

[14] Radloff LS (1977). The CES-D scale:a self-report depression scale for research in the general population. Appl Psychol Meas.

[15] Folstein MF, Folstein SE, McHugh PR (1975). "Mini-mental state". A practical method for grading the cognitive state of patients for the clinician. J Psychiatr Res.

[16] Benjamini Y, Hochberg Y (1995). Controlling the false discovery rate: a practical and powerful approach to multiple testing. J R Stat Soc Ser B Methodol.

[17] Cao YJ, Mager DE, Simonsick EM (2008). Physical and cognitive performance and burden of anticholinergics, sedatives, and ACE inhibitors in older women. Clin Pharmacol Ther.

[18] Hilmer SN, Mager DE, Simonsick EM (2009). Drug burden index score and functional decline in older people. Am J Med.

[19] Gnjidic D, Cumming RG, Le Couteur DG (2009). Drug burden index and physical function in older Australian men. Br J Clin Pharmacol.

[20] Gnjidic D, Bell JS, Hilmer SN (2012). Drug burden index associated with function in community-dwelling older people in Finland: a cross-sectional study. Ann Med.

[21] Lowry E, Woodman RJ, Soiza RL (2012). Drug burden index, physical function, and adverse outcomes in older hospitalized patients. J Clin Pharmacol.

[22] Wilson NM, Hilmer SN, March LM (2011). Associations between drug burden index and falls in older people in residential aged care. J Am Geriatr Soc.

[23] Nishtala PS, Narayan SW, Wang T (2014). Associations of drug burden index with falls, general practitioner visits, and mortality in older people. Pharmacoepidemiol Drug Saf.

[24] Jamieson HA, Nishtala PS, Scrase R, et al. Drug burden and its association with falls among older adults in New Zealand: a National Population Cross-Sectional Study. Drugs Aging. 2017. 10.1007/s40266-017-0511-5 29222667

[25] Gnjidic D, Hilmer SN, Blyth FM (2012). High-risk prescribing and incidence of frailty among older community-dwelling men. Clin Pharmacol Ther.

[26] Bosboom PR, Alfonso H, Almeida OP (2012). Use of potentially harmful medications and health-related quality of life among people with dementia living in residential aged care facilities. Dement Geriatr Cogn Dis Extra.

[27] Harrison SL, Kouladjian O'Donnell L, Bradley CE, et al. Associations between the drug burden index, potentially inappropriate medications and quality of life in residential aged care. Drugs Aging. 2018. 10.1007/s40266-017-0513-3 29322470

[28] Howel D (2012). Interpreting and evaluating the CASP-19 quality of life measure in older people. Age Ageing.

[29] Moriarty F, Cahir C, Bennett K (2017). Potentially inappropriate prescribing and its association with health outcomes in middle-aged people: a prospective cohort study in Ireland. BMJ Open.

[30] Gnjidic D, Hilmer SN, Hartikainen S (2014). Impact of high risk drug use on hospitalization and mortality in older people with and without Alzheimer's disease: a national population cohort study. PLoS One.

[31] Lonnroos E, Gnjidic D, Hilmer SN (2012). Drug burden index and hospitalization among community-dwelling older people. Drugs Aging.

[32] Best O, Gnjidic D, Hilmer SN (2013). Investigating polypharmacy and drug burden index in hospitalised older people. Intern Med J.

[33] Xue QL (2011). The frailty syndrome: definition and natural history. Clin Geriatr Med.

[34] Sexton E, King-Kallimanis BL, Layte R (2015). CASP-19 special section: how does chronic disease status affect CASP quality of life at older ages? Examining the WHO ICF disability domains as mediators of this relationship. Aging Ment Health.

[35] Richardson K, Kenny RA, Peklar J (2013). Agreement between patient interview data on prescription medication use and pharmacy records in those aged older than 50 years varied by therapeutic group and reporting of indicated health conditions. J Clin Epidemiol.

[36] Nishtala PS, Hilmer SN, McLachlan AJ (2009). Impact of residential medication management reviews on drug burden index in aged-care homes: a retrospective analysis. Drugs Aging.

[37] Gnjidic D, Le Couteur DG, Abernethy DR (2010). A pilot randomized clinical trial utilizing the drug burden index to reduce exposure to anticholinergic and sedative medications in older people. Ann Pharmacother.

